# The impact of community midwives on maternal healthcare utilization

**DOI:** 10.1002/hec.4640

**Published:** 2022-12-01

**Authors:** Tareena Musaddiq

**Affiliations:** ^1^ Ford School of Public Policy University of Michigan Michigan Ann Arbor USA

**Keywords:** community health workers, developing countries, maternal health, midwives, skilled birth attendants

## Abstract

Globally 800 women die every day from preventable causes related to pregnancy and childbirth. One of the major reasons for high maternal mortality ratios in many developing countries is the low proportion of births attended by Skilled Birth Attendants (SBA). To address the high number of maternal deaths, in 2008 the Government of Pakistan introduced the Community Midwives Program. Under the program, women from across the country were trained and deployed as Community Midwives. In this study, I use six rounds of Pakistan Social and Living Measurement Survey to estimate the impact of this program on maternal healthcare utilization. I find that women residing in districts with higher Community Midwives per capita were 9 percentage points more likely to be attended by a SBA at the time of delivery and were 8 percentage points more likely to give birth at a medical facility as opposed to birthing at home. I find no evidence of impact on take up of prenatal and post‐natal check‐ups. The use of Community Midwives may be a cost effective tool to reduce maternal deaths, especially for developing countries with low health budgets.

## 1 INTRODUCTION

### 1.1 Motivation

Globally 800 women die every day from preventable causes related to pregnancy and childbirth. Ninety four percent of maternal deaths worldwide are from developing countries, with one in every five occurring in the South Asian region.^1^ For Pakistan, the fifth most populous country in the world, the Maternal Mortality Ratio stands at 178 deaths per 100,000 live births compared to just 12 in the developed world (World Health Organization, 2019). Half of all maternal deaths worldwide occur in six countries; Pakistan is one of them (Hogan et al., 2010).

High maternal mortality rates in developing countries such as Pakistan are associated with low proportion of deliveries attended by Skilled Birth Attendants (SBA) (Girum & Wasie, 2017). Out of an estimated 4‐5 million births per year in Pakistan, less than one in every two is attended by a skilled health personnel.^2^ The rate of pre‐natal checkups is also low, with less than 40% of women receiving four or more pre‐natal checkups, and one in every three receiving no pre‐natal care at all (PDHS, 2007).

To address this issue of low utilization, and the broader goal of reducing maternal deaths, the Government of Pakistan drafted the Maternal Newborn and Child Health (MNCH) Policy in 2005. One of the major tools employed was the introduction of Community Mid‐Wives (CMWs) as front line health workers. In this study, I estimate the impact of deploying CMWs on maternal healthcare utilization at the time of giving birth, as well as during pregnancy and post‐partum.

### 1.2 The Pakistani context

The Pakistan Demographic and Health Survey (PDHS) of 2006–07 shows that pregnancy related illnesses are the leading cause of death for women of childbearing age (PDHS, 2007). Pakistan ranked 149, out of 179 countries in the world, on the Mother's Index Rank in the year 2015 (Save the Children, 2015). The state of maternal health in Pakistan may be attributed to both demand and supply side problems. The low demand for professional maternal health care is associated with low female literacy, lack of trust in medical professionals and preference for local traditional practitioners, and low mobility of women. On the supply side, lack of healthcare facilities that provide appropriate pre‐natal care, limited access to emergency obstetric and newborn care and lack of SBAs are considered the main reasons for high maternal deaths (Technical Resource Facility, 2013).

Pakistan's MNCH policy of 2005–06 was designed to improve the high maternal, infant and child mortality rates in the country, with the aim of bringing these numbers in line with the Millennium Development Goals. In particular, the program aimed to achieve these goals by increasing utilization of pre‐natal care, institutional births, and presence of skilled attendants at birth. One of the major tools employed was the introduction of CMWs as front line health workers, a concept new to Pakistan's healthcare system. The program was inspired by Indonesia's Village Midwife Program of early 1990s, which introduced more than 50,000 midwives in rural areas of Indonesia (Shrestha, 2010). The Pakistan government envisioned the program to expand up to one CMW per 5000 of the population in the very long term, with a short term goal of each CMW being assigned to a catchment area of 10,000 (Government of Pakistan, 2006).

The eligible candidates for CMW training were females aged 18–35 years who had cleared the national level matriculation exam (10^th^ grade). Women from all regions of the country were to be recruited, trained and deployed back in areas of their residence and origin, where they would have both the knowledge about and trust of the local communities, making it easier for the local residents to accept their role. The selected candidates were trained in‐class for a year at one of the certified institutes of Pakistan Nursing Council, followed by 6 months of field training.^3^

Pakistan, on average, invests less than 4.2% of its overall budget in health, which is low compared to other comparable countries in the region such as Sri Lanka (11.17%), Indonesia (6%), Bangladesh (5.7%), and India (5%).^4^ Training and deploying CMWs is a relatively low cost solution to address the problem of maternal health and increase the number of skilled front line workers to assist in deliveries. Community Mid‐Wives can be trained in a much shorter time (18 months) and at a lower cost in comparison to doctors and nurses. Other developing countries like Indonesia, Sri Lanka and Sierra Leon, have in the past used CMWs to address maternal and infant health.^5^

While inherently a supply side intervention, the policy also catered to barriers in demand by ensuring CMWs were placed back in areas of their origin upon successful completion of training. Being accustomed to the local language, culture and community would help the CMWs in reaching out to women in their areas and the familiarity would make the local community more receptive to them. Upon deployment, their role was to provide individualized care to the pregnant women throughout the maternity cycle and subsequently to the newborn. It was expected that the interaction of the local women with CMWs would change health‐seeking behaviors by impacting the take up of pre‐ and post‐natal visits, and the rate of skilled birth attendance. Additionally, the CMWs were trained to identify actual or anticipated conditions requiring medical attention and make timely referrals of obstetric and newborn complications, bringing down the number of deaths due to untreated complications.^6^

In terms of roll out and implementation, deployment of CMWs to their respective catchment areas started in 2008. By 2013, 45% of the planned 12,000 CMWs were trained, out of which 64% were deployed successfully (Technical Resource Facility, 2013).

### 1.3 Existing evidence on community health workers and community MidWives

Community Health WorkersCHW are defined as members of the community who are trained to promote health or carry out some healthcare services (Lipp, 2011). These workers traditionally act as a bridge between the health care delivery system and the community, leveraging their ability to help others due to their knowledge of the communities they serve and the close bond they can form with them (Kowitt et al., 2015). Recently, there has been a renewed interest in CHWs. In particular, developing countries are interested in leveraging the cost effectiveness and outreach of these workers to remote areas. Community Mid‐Wives can be trained in a much shorter time and at lower cost than doctors or nurses. Community Mid‐Wives are employed for various purposes. Improving maternal, infant and child health, increasing take up of immunizations and family planning services, promoting health seeking behavior, and awareness of diseases such as human immunodeficiency virus (HIV) and malaria are some of the most popular ones. In conservative regions, like Pakistan, where women are traditionally restricted in physical movement, female workers are often effective in reaching out to women who otherwise have limited access to healthcare services (Ayiasi et al., 2015; Bolton, Bass & Neugebaur, 2003; Rahman et al., 2008). Numerous studies find strong support for CHWs, in terms of their association with better health behaviors and outcomes. In the U.S., they have been used to target disadvantaged communities as enablers of HIV testing (Massnegale et al., 2016), health care of aging populations (Kaur, 2016), chronic disease measurement (Kim et al., 2016), and asthma interventions in children (Stephens et al., 2009), among others. In the developing world, CHWs are found to be positively associated with breast‐feeding practices, family planning, antenatal care, neonatal check‐ups, and immunizations (Corluka et al., 2009; Lipp, 2011; Omer et al., 2008).

In the early 1990s, Indonesia introduced a special cadre of CHWs, Community Midwives (CMWs), to deal with high fertility, maternal mortality and infant mortality rates. Over 50,000 CMWs were introduced in rural areas of Indonesia and the evidence on the effectiveness of the intervention is mixed. Studies have shown the program was ineffective in terms of increasing use of contraception (Weaver et al., 2013). Others find that while the deployment of a mid‐wife in every village increased skilled birth attendance, it did not lead to timely referrals to specialized obstetric care for those in need of it (Ronsman et al., 2001). Evidence from an evaluation of a similar program in Bangladesh showed reduced rates of maternal mortality from training and deploying midwives at the village level (Fauveau et al., 1991).

Studies evaluating the CMW program of Pakistan largely use qualitative techniques such as focus group discussions and interviews to assess outreach and effectiveness (See Noorani et al., 2013, Sarfraz & Hamid, 2014, Ahmed et al., 2017). Other research evaluates the equity, class and social exclusion aspect of the program in selected districts (Mumtaz et al., 2012). These studies provide important insights about barriers to outreach and the effectiveness of the program. They are, however, often geographically restricted to one or two districts of the country, limiting the scope of their findings to the districts evaluated (e.g., Mumtaz et al., 2012 focus on two districts in Punjab, Ahmed et al., 2017 focus on 2 districts each in Punjab and Khyber Pakhtunkhwa).

### 1.4 Purpose of the study & contribution

In this study, I pool 6 years of cross‐sectional household surveys in Pakistan to quantitatively analyze the impact of deploying CMWs on maternal health care utilization. I make use of information on maternal health care from over 90 districts in the country to assess the average impact at the national level. The main outcomes of interest are any pre‐natal checkup, any post‐natal checkup, whether a skilled birth attendant helped deliver the baby and whether the women gave birth at a medical facility (as opposed to giving birth at home).

This, to my knowledge, is the first attempt to study the impact of the program at a national scale. As discussed in the literature review, only a few studies around the world use data to quantitatively assess the impact of frontline, low cost health workers. This study bridges the gap by evaluating the effectiveness of such workers for improving maternal health in developing countries and focuses on Pakistan‐a country with one of the highest number of maternal deaths worldwide.

From a policy perspective, the study evaluates whether the deployment and training of CMWs is an effective policy tool for encouraging women to seek maternal health care during pregnancy (any pre‐natal and post‐natal check‐up) and at the time of giving birth (presence of a SBA and birth at a medical facility). Results of the study provide information on whether this cadre of health workers may help in improving maternal healthcare utilization.

## 2 METHODS

### 2.1 Data

The study primarily employs the Pakistan Social and Living Measurement Survey (PSLM). The PSLM is a cross sectional survey conducted each year across the country, alternating between being representative at the district or provincial level.^7^ The survey focuses on measuring progress toward the Millennium Development Goals (MDGs), along with detailed sections on employment and household wealth. The universe for the survey consists of all urban and rural areas of the main provinces of Pakistan, excluding districts that are part of the Federally Administered Tribal Areas and military restricted areas, which comprise 2% of the overall population. The data were obtained from the Pakistan Bureau of Statistics.

For this study, I use six years of the district level representative data sets for the years between 2004 and 2014. In each of these rounds approximately 75,000 households and their members were interviewed on questions related to employment, education, water and sanitation, household wealth and health, with specific sections on the health of children, and women of childbearing age. In addition, questions pertaining to health utilization and satisfaction regarding available health services were also included in the survey. The relevant outcomes of interest for this study come from the section on women's health, which is administered to women who had given birth in the 3 years prior to the survey. Details of the total sample size of PSLM and the sample of women surveyed for maternal health related questions, over the six rounds used for estimation purposes in this study, are provided in Table A1.

I supplement this main data set with data on the deployment of CMWs at the district level. These data were obtained from the MNCH website.^8^ The data provide information on the number of midwives deployed, disaggregated by district. Statistics related to population and growth rates of population, for per capita calculations, were obtained from the World Bank and reports from the Census of Pakistan 1998.^9^

Summary statistics for variables used in the study are shown in Table 1 for pre and post treatment time periods.^10^ Since districts receive the treatment at the same point in time (i.e., 2008), I use the variation in “dose” of the treatment across districts to estimate the impact of the treatment. The average number of CMWs in a district is around 35, while 22 out of the 99 districts had no CMWs deployed. Districts in Pakistan differ widely in population size. I therefore use CMW per 10,000 of the population as the main variable of interest. On average, 0.16 CMWs were deployed per 10,000 of the population as a result of the program. Details on the average number of CMWs per 10,000 of the population in each district are provided in Table A2.

**TABLE 1:** Summary statistics

	Mean		Difference	Min	Max
Variable	Pre	Post	(Post‐pre)		
Treatment					
*CMW_d_* (total)	‐	35.18	‐	0	109
*CMW_d_* (per 10,000 of the population)	‐	0.161	‐	0	0.860
Outcomes of interest					
Pre‐natal checkup	0.510	0.610	0.097***	0	1
Institutional birth	0.287	0.402	0.111***	0	1
Skilled birth attendant	0.423	0.450	0.024***	0	1
Post‐natal check up	0.237	0.266	0.028***	0	1
Demographics & HH characteristics					
Age	28.95	28.95	0.002	16	50
Education of the HH head	4.800	4.802	−0.003	0	21
No. Of members in the HH	8.324	7.978	−0.346***	2	20
Real HH income (in 000 PKR.)^20^	8.672	16.774	8.148***	1.50	500
TV ownership	0.445	0.507	0.062***	0	1
House ownership	0.870	0.695	0.175***	0	1
Urban residence	0.359	0.462	0.103***	0	1
Access to healthcare					
Time to nearest health facility 0–14 min.	0.402	0.490	−0.027***	0	1
Time to nearest health facility 15–29 min.	0.251	0.282	−0.031***	0	1
Time to nearest health facility 30–44 min.	0.187	0.162	0.023***	0	1
Time to nearest health facility 45–59 min.	0.054	0.051	0.003**	0	1
Time to nearest health facility 60+ mins.	0.106	0.074	0.032***	0	1
LHW visit in last 30 days	0.511	0.586	0.075***	0	1
Observations	54,093	116, 266			

*Note*: The pre‐period data is from the 2004 and 2006 rounds of PSLM. The post period data is from 2008, 2010, 2012 and 2014 rounds of PSLM. Pre‐natal check is a binary indicator equal to one if the woman had at least one prenatal check‐up during pregnancy, and zero otherwise. Post‐natal check is a binary indicator equal to one if the woman had a postnatal check‐up within 6 weeks of delivering the child, and zero otherwise. Skilled Birth Attendant is a binary indicator equal to one if the woman delivered the child in the presence of a skilled birth attendant, and zero otherwise. Institutional delivery is a binary indicator equal to one if the woman delivered the child in the medical facility, and zero if the woman delivered the child home. Detailed definitions of other variables are provided in Table A3.

Abbreviations: CMW, community mid‐wives; HH, household; LHW, lady health worker; PKR, Pakistani rupee; TV, television.

***p < 0.01, **p < 0.05, *p < 0.1.

**FIGURE 1:**
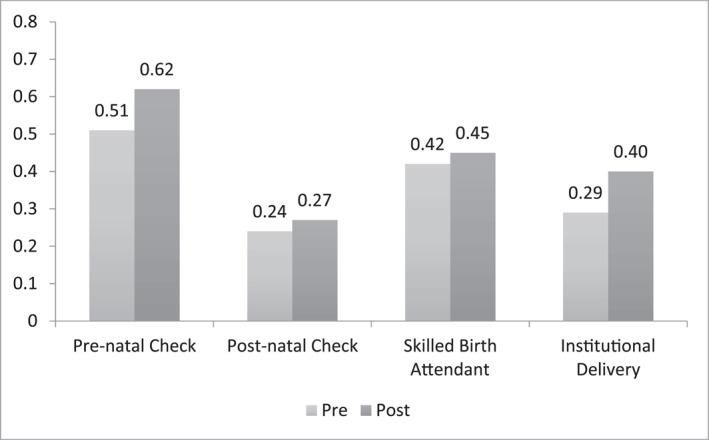
Rates of maternal healthcare utilization. The pre‐period data is from the 2004 and 2006 rounds of Pakistan Social and Living Measurement Survey (PSLM). The post period data is from 2008, 2010, 2012 and 2014 rounds of PSLM. Pre‐natal check is a binary indicator equal to one if the woman had at least one prenatal check‐up during pregnancy, and zero otherwise. Post‐natal check is a binary indicator equal to one if the woman had a postnatal check‐up within 6 weeks of delivering the child, and zero otherwise. Skilled Birth Attendant is a binary indicator equal to one if the woman delivered the child in the presence of a skilled birth attendant, and zero otherwise. Institutional delivery is a binary indicator equal to one if the woman delivered the child in the medical facility, and zero if the woman delivered the child home. *Y*‐axis shows the average of the variable

**FIGURE A1:**
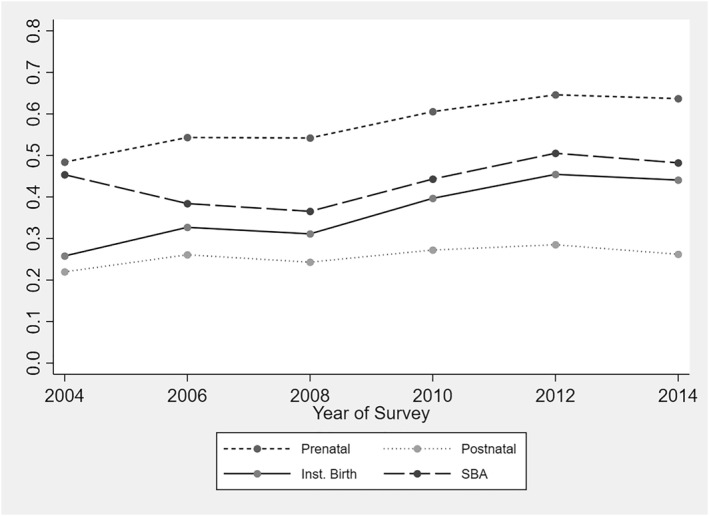
Trends in Maternal Healthcare Utilization in Pakistan. Data comes from six rounds of Pakistan Social and Living Measurement Survey (PSLM survey. Sample is women of childbearing age who had given birth in the last 3 years prior to the survey. *X*‐axis shows the year of survey. *Y*‐axis shows the average rate of maternal healthcare utilization. Prenatal is a binary indicator equal to one if the woman had *at least* one prenatal check‐up during pregnancy, zero otherwise. Postnatal is a binary indicator equal to one if the woman had a postnatal check‐up within 6 weeks of delivering the child, zero otherwise. SBA (Skilled Birth Attendant) is a binary indicator equal to one if the woman delivered the child in the presence of a skilled birth attendant, zero otherwise. Institutional birth is a binary indicator equal to one if the woman delivered the child at a medical facility/institution, zero if delivered at home

The relevant sample for this study is women who were in the childbearing age (16–50 years) at the time of survey and had given birth in the last three years. This criterion yields a sample size of more than 173,000 observations over six rounds of the survey. The four outcomes of interest are binary indicators for (i) pre‐natal checkup, (ii) post‐natal checkup, (iii) birth attended by a SBA and (iv) birth at a medical institution. Table 1 shows each of these measures are statistically different in the pre and post periods. Figure 1 shows the distribution of binary responses for each of these outcomes of interest in the pre‐ and post‐treatment periods. In the pre‐treatment period, one in every two women did not have *any* pre‐natal check‐ups. In the post period a larger proportion of women (61%) report having at least one pre‐natal check‐up during pregnancy. The proportion of women who underwent a post‐natal examination increases from 24% in the pre period to 27% in the post period. In terms of healthcare at the time of delivery, only 42% women had a skilled birth attendant assisting the delivery in the pre‐treatment period. This proportion increased to 45% in the post‐treatment period. We see a big change in the proportion of births occurring at a medical facility (as opposed to home). While only 29% women gave birth at a medical facility in the pre period, 40% of women report delivering at a medical institution in the post period; a change of 11% points. Figure A1 tracks the outcomes variables over the 6 rounds of survey. CMWs were deployed in 2008. Post the deployment of the CMWs, higher rates of maternal healthcare utilization were reported in the subsequent rounds of the survey.

In terms of access to health services, Table 1 shows, 40% of households in the sample report living within 15 min of travel time to a health facility.^11^ Over half of the households report that a Lady Health Worker (LHW) visited their house in the 30 days prior to the survey. The role of LHWs in Pakistan is to provide preventative and curative health services to members in their assigned community through home visits. Like CMWs, their peer status enables them to connect with patients and navigate local customs, languages, and social relationships more effectively than outsiders. Their duties include creating awareness of and providing information on reproductive health, nutrition and hygiene, facilitating registrations of births and deaths, distributing medication for family planning and immunizing children. LHWs act as liaisons between the formal health system and their community by encouraging uptake of public health initiatives and referring patients to nearby clinics (Zhu et al., 2014). Community Mid‐Wives, unlike LHWs, are medically trained to provide care during pregnancy and childbirth, identify complications during pregnancy, help deliver the child and provide post‐natal care.

Statistics in Table 1 also describe the characteristics of the women in the sample and of their households. The average woman in the sample is 29 years of age. Head of the household on average has 4.8 years of education. Each household has around 8 members. There is a significant increase in the proportion of households residing in urban areas between the pre and post period; 45% in post period compared to 36% in the pre‐period. The household income and wealth indicators are also substantially higher on average in the post treatment period. The change in household income is consistent with the increase in real gross domestic product (GDP) per capita for Pakistan for the time period over which the data spans.^12^ I control for all these variables in my estimations to account for any difference in outcomes that might be due to these factors.

### 2.2 Empirical methodology

#### 2.2.1 Testing for endogeneity

The Government of Pakistan implemented the policy at the national level, with varying dose of treatment for each district. There is, however, concern that the treatment may be endogenous. For example, if districts with worse maternal health care receive a higher dose of the treatment, the estimate of the impact will likely be biased. To address this concern, I follow the approach taken by Bollen et al. (1995) to account for endogeneity. I test whether district characteristics predict the treatment intensity. I aggregate the maternal healthcare measures and economic indicators up to the district level using pre‐period rounds of the survey (2004 and 2006), and model the treatment variable (CMW per 10,000 of the population) on *pre‐period* averages of these variables, using Ordinary Least Squares as follows:

(1) $${\mathrm{CMW}}_{d}{=\alpha }_{o}+\alpha_{1\;}{\mathrm{EC}}_{d}+\alpha_{2}{\mathrm{MH}}_{d}+\varepsilon_{d}$$

where *CMW*_*d*_ is the treatment variable defined as the average number of CMWs per 10,000 of the population in district *d*, *EC*_*d*_ represents a vector of controls capturing the average economic conditions of households in the districts in the pre‐period, *MH*_*d*_ represents a vector of controls capturing average pre‐period rates of maternal health practices and $\varepsilon_{d}$ represents the random error term.^13^ Averaging variables at the district level results in one observation per district for this estimation.

I test whether district characteristics in the pre‐period (averaged using 2004 and 2006 rounds of the PSLM survey) predict the deployment of CMWs. I test whether the average rates of the main outcomes of interest (pre‐ and post‐natal check‐ups, skilled birth attendance and institutional birth) in the years prior to the introduction of CMWs predict the number of CMWs deployed per 10,000 of the population in the district. In addition, I check whether other characteristics such as the economic well‐being of the district (average district income, proportion of household (HHs) with television (TV), proportion of rural HHs) and outreach of existing health facilities (proportion of HHs visited by LHWs) are predictive of the treatment.

Table 2 shows the results from estimating Equation (1). If the policy was implemented in a way most beneficial for districts most in need, we would expect the pre‐period district characteristics to predict deployment of CMWs per 10,000 of the population, in turn implying that the estimate of $\beta_{1}$ from Equation (2) would be biased. In Table 2, however, we see that pre‐period measures of maternal healthcare utilization do not predict CMWs per 10,000 of the population in a district (*p*‐value of 0.52 on test of joint significance).^14^ Further, other economic and social characteristics of the district are also not significantly correlated with CMW per 10,000 of the population (*p*‐value of 0.58 on joint test of significance).^15^ Finally, both the maternal healthcare utilization measures and other district characteristics jointly also do not predict the treatment (*p*‐value of 0.41 on joint test of significance).^16^ Proportion of households in the district that received a visit from the LHW in last 30 days is the only variable significantly correlated with CMWs per 10,000 of the population. This might indicate that districts with better infrastructure were targeted more by the policy, possibly owing to ease of implementation, as opposed to the need. Households residing in districts with better health facilities and outreach of LHWs might be more receptive to the CMW program, since the major responsibility of LHWs is to visit households to educate people about health services and promote health seeking behaviors. To account for this, I control for a LHW visit to the household in the last 30 days in my main estimation equation (see Equations (2) and (3) below), among other controls.

**TABLE 2:** Predicting deployment of Community Mid‐Wive (CMW) in the district

	CMWs per 10,000
Proportion of rural HHs in the district	0.278
	(0.210)
Average HH income (PKR 000)	0.00547
	(0.00937)
Proportion of women who had a post‐natal check up	3.052
	(3.499)
Proportion of women who had a pre‐natal check up	−2.228
	(2.939)
Proportion of institutionalized births	−3.633
	(5.836)
Proportion of births with skilled attendance at birth	6.599
	(5.621)
Proportion of HHs that own a TV	0.0302
	(0.127)
Proportion of HHs that received a LHW visit	0.236***
	(0.0812)
Observations	99
R‐squared	0.207

*Note*: Results are from estimation of Equation (1). All variables are averaged up to the district level. Outcome variable is the average number of CMWs per 10,000 of the population deployed in the district. The independent variables are from the pre‐periods (2004–05 and 2006–07 rounds of PSLM). Robust standard errors in parentheses.

Abbreviations: HH, household; LHW, lady health worker; PKR, Pakistani rupee; TV, television.

***p < 0.01, **p < 0.05, *p < 0.1.

Next, following Bollen et al. (1995), I predict residuals from estimation of Equation (1) and test whether the residuals predict the main outcomes of interest. Equation (2) below shows the main equation of interest with residuals added as an explanatory variable.

(2) $$\mathrm{Pr}\left(y_{id}=1\right)=\Phi \;\left(\theta_{0}+\theta_{1}{\mathrm{CMW}}_{d}+\theta_{2}X_{id}+\theta_{3}Z_{id}+{\theta_{4}{Res}_{d}+\delta }_{t}+\varepsilon_{id}\right)$$

where i indicates the individual, and d indicates districts. $y_{id}$ is the outcome of interest (e.g., binary indicator for pre‐natal checkup). *CMW*_*d*_ is the continuous treatment variable defined as CMWs deployed per 10,000 of population in district *d*, taking on a value of zero for pre‐treatment periods. *X*_*id*_ are individual and household level controls while *Z*_*id*_ are other controls related to the area of residence and the available health facilities, and $\delta_{t}$ are survey year fixed effects (for detailed definitions of variables see Table A3). ${Res}_{d}$ are predicted residuals from estimation of Equation (1). A statistically insignificant coefficient on these residuals ($\theta_{4})$ in estimation of Equation (2) would imply that deployment of CMW per 10,000 of the population is conditionally exogenous and treatment selection is not endogenous. Note that residuals are predicted at the district level and Equation (2), therefore, does not control for district fixed effects.

Table A5 shows the results from probit regression of Equation (2) using each of the four measures of maternal healthcare utilization as the outcome. In each of the estimation results in Panel A, the coefficient on the predicted residuals ($\theta_{4})$ is statistically insignificant, allowing us to assume that the treatment (and the intensity of the treatment) is not endogenous.

#### 2.2.2 Estimating the impact of Community Mid‐Wives on maternal healthcare utilization

I use a difference‐in‐differences approach with a continuous treatment variable and use variation in treatment intensity to evaluate the effect of the program. More precisely, I use the difference in “dose” of the treatment, measured by CMWs deployed per 10,000 of the population in the district, to determine the impact of the program using pre‐ and post‐period data. The main outcomes of interest are binary and are defined as: (i) having undergone at least *one* prenatal checkup during pregnancy, (ii) having a post‐natal checkup within 6 weeks of delivery, (iii) having a skilled birth attendant present at the time of delivery and, (iv) delivering the baby at a medical facility (i.e., institutional birth).

The probit model estimated for the main outcomes of interest is as follows:

(3) $$\mathrm{Pr}\left(y_{id}=1\right)=\Phi \;\left(\beta_{0}+\beta_{1}{CMW}_{d}+\beta_{2}X_{id}+\beta_{3}Z_{id}+{\gamma_{d}+\delta }_{t}+\varepsilon_{id}\right)$$

where $\gamma_{d}$ are district fixed effects and all other variables are as defined for Equation (2) above. The district fixed effects account for time‐invariant differences across districts, including pre‐period differences across districts in maternal healthcare utilization and socioeconomic characteristics. $\beta_{1}$ estimates the impact of an additional CMW per 10,000 of the population. A positive and statistically significant value of $\beta_{1}$ would imply that the program increased maternal healthcare utilization (as measured by the outcome).

## 3 RESULTS

### 3.1 Impact of Community Mid‐Wives on maternal healthcare utilization

I estimate the impact of CMWs on maternal health care utilization using individual level micro data from six rounds of the PSLM. Table 3 shows the results from estimating Equation (3).^17^ Panel B of Table 3 shows the corresponding marginal effects of *CMW*_*d*_ for estimations in Panel A.

**TABLE 3:** Impact of Community Mid‐Wives (CMWs) on maternal health care utilization

	1	2	3	4
	Pre‐natal	Post‐natal checkup	SBA	Inst. Birth
Panel A: Probit Estimates				
CMW per 10k	−0.045	0.10	0.271**	0.257**
	(0.151)	(0.231)	(0.080)	(0.081)
Panel B: Marginal Effects				
CMW per 10k	−0.015	0.030	0.089***	0.080***
	(0.049)	(0.069)	(0.026)	(0.0248)
Mean of the outcome	0.58	0.26	0.44	0.36
Observations	170,359	170,359	170,359	170,359

*Note*: Sample is women who had given birth in the last 3 years prior to the survey. Panel A shows results from probit estimation of Equation (3). Panel B shows the corresponding marginal effects from the estimations in Panel A. CMWs per 10,000 of the population in the district is the treatment. All estimations have the following controls: age dummies, education of the household head, real household income, number of members in the households, indicators for wealth of the household, indicator for rural/urban locality, indicators for time to nearest health facility, district fixed effects and survey year fixed effects. SBA stands for “skilled birth attendant”. Standard errors are clustered by district.

***p < 0.001, **p < 0.01, *p < 0.05.

I begin by looking at utilization behavior prior to and after delivery. In Columns 1 and 2, I find no evidence of a significant impact of CMW's deployment in the district on the probability of women seeking pre‐ and post‐natal check‐ups. The coefficients though positive are imprecisely estimated.

Next, I look at the impact of CMWs on whether the women are attended by a SBA at the time of delivering the child. A positive coefficient in Column 3 of Table 3 shows that women are more likely to have a SBA at the time of birth if there are more CMWs per 10,000 available in their district. With an average of 0.16 CMWs per 10,000 introduced by the program, the marginal effect estimates (Table 3, Panel B) imply that as a result of the program women were on average 1.28% points more likely to give birth at a medical facility and 1.44% points more likely to be attended by a SBA at the time of birth.

Table 4 shows the coefficients for all variables in Equation (3), including the controls. The results show some interesting relationships. First, having a more educated head of the household and a higher income for the household, is positively associated with all four outcomes of interest. This suggests that demand side may be at play and developing policies aimed at informing the less educated households about the importance of these services may be helpful. Second, on the supply side, in terms of access we see a consistent pattern for all outcomes. The longer it takes to reach the nearest health facility, individuals are less likely to utilize any maternity related service. This has two important implications. First, health workers like LHWs who are assigned responsibility to visit households are of great importance especially for households that are away from medical facilities like Basic Health Units or Rural Health Units. While individuals in these households are less likely to go out and seek services, providing services closer to home might help change health behaviors. For CMWs as well, it may be more important to reach out to households that are a considerable distance away from health facilities.

**TABLE 4:** Results of probit estimations

	(1)	(2)	(3)	(4)
	Pre‐natal	Post‐natal	SBA	Inst. Birth
CMW	−0.045	0.099	0.271***	0.257***
	(0.151)	(0.231)	(0.0801)	(0.0806)
Urban	0.126***	0.134***	0.210***	0.171***
	(0.024)	(0.027)	(0.019)	(0.016)
LHW visit in last 30 days	0.085***	0.0352	0.0316	−0.0350
	(0.025)	(0.040)	(0.026)	(0.024)
Time to nearest health facility (15–29 min)	−0.129***	−0.0893***	−0.137***	−0.153***
	(0.021)	(0.028)	(0.019)	(0.017)
Time to nearest health facility (30–44 min)	−0.216***	−0.129***	−0.219***	−0.228***
	(0.025)	(0.034)	(0.023)	(0.023)
Time to nearest health facility (45–59 min)	−0.303***	−0.264***	−0.269***	−0.306***
	(0.043)	(0.042)	(0.039)	(0.033)
Time to nearest health facility (60+ mins.)	−0.381***	−0.363***	−0.325***	−0.347***
	(0.056)	(0.059)	(0.036)	(0.034)
HH head's education	0.031***	0.030***	0.0374***	0.040***
	(0.002)	(0.002)	(0.002)	(0.002)
HH income (PKR 000s)	0.006***	0.005***	0.008***	0.008***
	(0.001)	(0.0004)	(0.0001)	(0.0005)
No. of HH members	0.001	−0.007***	−0.009***	−0.010***
	(0.002)	(0.002)	(0.002)	(0.002)
TV ownership	0.274***	0.252***	0.301***	0.314***
	(0.017)	(0.018)	(0.018)	(0.020)
House ownership	0.047***	0.045**	−0.002	0.017
	(0.018)	(0.022)	(0.016)	(0.016)
Observations	170,359	170,359	170,359	170,359

*Note*: Sample is women who had given birth in the last 3 years prior to the survey. The results are from estimations of Equation (3). Variables are as defined in Table A3. Estimates for age dummies, survey year fixed effects and district fixed effects are excluded for brevity. SBA stands for “skilled birth attendant”. Standard errors are clustered by district.

Abbreviations: CMW, community mid‐wives; HH, household; LHW, lady health worker; PKR, Pakistani rupee; TV, television.

***p < 0.001, **p < 0.01, *p < 0.05.

Results in Table 4 also reflect the rural urban divide. Individuals residing in rural areas are less likely to seek maternal health care, even after controlling for other household and access variables. This may have implications for future deployment of such health workers.

### 3.2 Further estimations

Twenty two of the districts received no CMWs under the program. I exclude these districts from the sample and re‐estimate Equation (3). Results in Table 5 show that coefficients for CMWs per 10,000 of the population from probit estimations (and the corresponding marginal effects) for institutional births and SBA remain statistically significant and very close in magnitude to the full sample estimation in Table 3. This implies that the results in Table 3 were not entirely driven by differences in districts that did and did not receive the treatment and the “dose” of the treatment among districts that received the treatment is also important.

**TABLE 5:** Impact of Community Mid‐Wives (CMWs) on Maternal Healthcare Utilization (excluding districts with no CMWs)

	(1)	(2)	(3)	(4)
	Prenatal	Postnatal	SBA	Inst. Birth
Panel A: Probit Estimates				
CMW per 10k	−0.037	0.124	0.265**	0.251**
	(0.157)	(0.251)	(0.086)	(0.087)
Panel B: Marginal Effects				
CMW per 10k	−0.012	0.038	0.087**	0.079**
	(0.051)	(0.076)	(0.028)	(0.027)
Observations	146,024	146,024	146,024	146,024

*Note*: Results are from estimations of Equation (3), excluding the sample of women from districts where no CMWs were deployed. SBA stands for “skilled birth attendant”. All estimations have the following controls: age dummies, education of the household head, real household income, number of members in the households, wealth of the household, rural/urban locality, time to nearest health facility, district fixed effects and survey year fixed effects. Panel A shows the probit coefficients and Panel B shows the corresponding marginal effect. Standard errors in parentheses are clustered by district.

***p < 0.01, **p < 0.05, *p < 0.1.

The recall period for maternal healthcare related questions in the PSLM survey is 3 years. To address the concern of the 2008–09 round of PSLM overlapping with the year of the CMW intervention, I exclude the 2008–09 PSLM sample from my data and re‐estimate Equation (3). Results are shown in Table 6. Compared to results in Table 3, results remain robust with estimates changing only slightly.

**TABLE 6:** Impact of Community Mid‐Wives (CMWs) on maternal healthcare utilization (excluding the 2008–09 round of Pakistan Social and Living Measurement Survey (PSLM))

	(1)	(2)	(3)	(4)
	Pre‐natal	Post‐natal	SBA	Inst. Birth
Panel A: Probit Estimates				
CMW per 10k	−0.0856	0.0905	0.258***	0.263***
	(0.151)	(0.234)	(0.0849)	(0.0800)
Panel B: Marginal Effects				
CMW per 10k	−0.027	0.027	0.085***	0.082***
	(0.049)	(0.071)	(0.028)	(0.025)
Observations	140,759	140,759	140,759	140,759

*Note*: Results are for estimation of Equation (3). Sample excludes women from the 2008–09 round of the PSLM survey. All estimations have the following controls: age dummies, education of the household head, real household income, number of members in the households, wealth of the household, rural/urban locality, time to nearest health facility, district fixed effects and survey year fixed effects. SBA stands for “skilled birth attendant”. Panel A shows the probit coefficients and Panel B shows the corresponding marginal effects. Standard errors in parentheses are clustered by district.

***p < 0.01, **p < 0.05, *p < 0.1.

## 4 DISCUSSION

The basic aim behind the policy of deploying CMWs was to improve maternal health by increasing maternal healthcare utilization. This included pre and post‐natal checkups, as well as ensuring presence of skilled medical attendants at birth. Additionally, CMWs were expected to make timely referrals, as needed, to medical facilities where higher skilled personnel (e.g., doctors) would be available, and reduce the deaths due to undiagnosed or untreated complications.

The results in Table 3 imply that deployment of CMWs increases the probability of women having a skilled attendant at birth and the probability of giving birth at a medical institution (as opposed to delivering at home). The increased likelihood of giving birth at a medical institution may be explained in two ways. Community Mid‐Wives are provided resources by the Government under the MNCH policy to set up their own ‘clinic’ with ‘birthing stations.’ District Public Health Specialists and Lady Health Supervisors are assigned the role of visiting these birthing stations and ensuring their readiness to be operational.^18^ Zafar et al. (2015) show, in their mixed methods study, that almost half of women interviewed in rural areas of Pakistan expressed a preference for such birthing stations of CMWs, while only 20% preferred deliveries at home. Birthing stations were favored because of the availability of space and equipment and the proximity to their homes. This preference for birthing stations may explain the increase in institutional births post the deployment of CMWs. Referrals by CMWs in case of complications to medical facilities higher up in the health system, such as Basic and Rural Health Units (B.H.U and R.H.U) and hospitals, might also explain this positive impact. Analysis into breakdown of births within the different types of medical institutions and birthing stations may provide information on these channels, however the data used in this study does not allow such an analysis.

Results in Table 3 show no evidence of impact on utilization of pre‐ and post‐natal check‐ups. Coupled with a positive and large impact on giving birth at a medical institution, these results suggest that while utilization at the time of birth is improving, there is no evidence of change in the behavior regarding the care needed during pregnancy and in the period after childbirth. In terms of outreach, utilization of CMWs seems to have improved utilization at the time of birth but we do not find evidence of similar success in terms of pre‐ and post‐natal care.

This has several important implications. First, the point of having *community* midwives was to bridge any local and cultural barriers so that they are easily accepted in their roles by the local communities. However, it appears that despite increased contact with skilled birth attendants at the time of birth, women are not convinced about the importance of post‐natal checkups. Affordability is unlikely to be a barrier here since CMWs are allowed to charge only a very nominal fee from their patients and their basic compensation comes from the Government (Devlin et al., 2016).

A large part of the role outlined for CMWs is to provide individualized care during the entire course of the pregnancy. Lack of impact on pre‐natal checkups may signal deficiencies in outreach efforts of CMWs in terms of actively reaching out to pregnant women in their communities and making their presence and the services they offer more salient. If this is indeed the case, CMWs may need to be informed more clearly that their role is centered not only around delivering the child, but their services pre and post birth also have equal importance.

On the other hand, lack of impact of CMWs on pre‐natal and post‐natal care may also be explained by lack of understanding on the patients' end regarding the importance of pre and post‐natal checkups and the trust placed in CMWs (Khan & Khan, 2012). In more remote districts CMWs may be misunderstood as ‘doctors’ who need to be consulted in the event of a birth complication only (Mumtaz et al., 2012). In this case, policy requires that efforts be made to educate people about both the importance of pre‐ and post‐natal checkups, as well as toward building trust in reaching out to CMWs. Lack of information provision and advertisement might also be playing a role. Unfortunately given the data limitations, the channel that might be at play here cannot be explored in this study.

Existing initiatives, like LHWs who visit households, can be used to educate people about the importance and availability of the services offered by CMWs. In Table 4 we see no significant correlation of LHWs with three out of the four measures of maternal health care utilization. We, however, see a negative correlation with institutional births. This is a puzzling finding. However, some reports suggest that LHWs have not been used to refer patients to CMWs and medical health centers (Khan & Khan, 2012) and lack of coordination with other health providers may be one of the impediments to the success of this policy. The government can likely use LHWs to raise awareness about the availability of CMWs and the services they offer.

Further, research shows that most CMWs, being young and unmarried, are deemed as untrustworthy or inexperienced by the community with respect to their roles (Khan & Khan, 2012), in contrast to findings from remote districts where the opposite might be true (Mumtaz et al., 2012). Faisel (2012) reports that many CMWs feel their title does not depict their level of expertise, thereby limiting their acceptance. Efforts geared toward selling the role of CMWs to the local communities, beyond just deploying the CMWs back to their areas of residence may be needed.

## 5 CONCLUSION

Evidence on the effectiveness of initiatives for safe motherhood on health service use in developing countries is mixed. While it is generally agreed that skilled personnel should attend all births, the crucial question of where deliveries should take place and who qualifies as a skilled attendant remains a matter of debate. Nonetheless, indicators such as the proportion of births attended by skilled health personnel have gained credence, as is apparent by the push by the World Health Organization to set targets on proportion of births attended by skilled birth attendants.

In this paper, I evaluate the introduction of a new cadre of CMWs in Pakistan as frontline health workers aimed at impacting maternal health behaviors and increasing skilled attendance at birth. Findings from this study show CMWs positively impact skilled birth attendance and institutional births.

Approximately 2/3^rd^ of maternal deaths in developing countries occur in late pregnancy through 48 h after delivery (Nabudere et al., 2013). The four main causes of maternal deaths are obstructed labor, eclampsia, puerperal sepsis and obstetric hemorrhage, collectively accounting for 54% of maternal deaths in Pakistan (PDHS, 2007). SBAs, such as CMWs, are trained to identify and treat these causes and refer to higher skilled medical personnel when needed. Graham et al. (2001) show that these complications are preventable by 20–85% by SBAs depending on the complication and whether one takes a pessimistic or an optimistic estimate. The authors deduce a reduction of 16% to 33% in maternal mortality, assuming competent skilled attendants as well as an enabling environment for them to perform the necessary obstetric care‐both of which are provided under the CMW program of Pakistan as well. The cost of training and deploying a CMW in Pakistan is estimated at United States dollar (USD) 4710 and the cost of each subsequent delivery is USD 38.^19^ Estimates from this study show that each additional CMW per 10,000 of the population increases the likelihood of women being assisted at birth by a SBA by 9% points. Using Graham et al.’s (2001) estimates this implies a reduction in likelihood of maternal death by 1.44–2.97%.

I do not, however, find any evidence of impact on pre and post‐natal checkups. Further research is needed to evaluate whether measures such as enhancing the trust of the community and forming better linkages between other health initiatives (e.g., LHW) and facilities (e.g., linking CMWs to R.H.Us) may potentially improve pre and post‐natal check‐ups as well. Increase in post‐natal check‐ups may potentially also lead to gains in infant health as well, since CMWs are also trained to provide newborn care. Further research may help inform policy makers how the ultimate goal of reducing maternal and infant mortality was impacted by the CMWs. This study is also limited in not being able to speak to the possible mechanisms which might be leading to a significant impact on utilization at the time of birth but not on pre and post‐natal check‐ups. With more informative data, these questions may be answered to provide insights for better future implementation.

## CONFLICT OF INTEREST

The author declares that there is no conflict of interest that could be perceived as prejudicing the impartiality of the research reported.

## ETHICS STATEMENT

This study received the designation of ‘Not Human Subject Research’ from the Institutional Review Board of Georgia State University.

## Data Availability

The data that support the findings of this study are available from the corresponding author upon reasonable request.

## ENDNOTES

^1^

http://www.who.int/mediacentre/factsheets/fs348/en/.

^2^
The number of births is based on the birth rate according to www.data.worldbank.org. The rate of skilled birth attendance is based on the data used in this study (See Table 1).

^3^
See Appendix B for details of the program.

^4^
Data retrieved from http://apps.who.int/gho/data/.

^5^
See Weaver et al. (2013); Ngongo et al. (2013); Siriwardhana et al. (2019).

^6^
For details on the aim and objectives of the CMW program see Appendix B. These details are based on the information on the Maternal Newborn and Child Health Program (PC‐1) document (Government of Pakistan, 2006) and the Pakistan MNCH website.

^7^
District is the third tier of governance in Pakistan, while province is the second.

^8^

http://dynasoft.org/mnch/.

^9^

http://data.worldbank.org/indicator/.

^10^
For detailed description of the variables see Table A3.

^11^
Nearest Health Facility includes Basic Health Unit, Rural Health Unit, Hospital or a Doctor's Clinic.

^12^
Between 2004 and 2014 real GDP per capita in Pakistan increased by more than $150 (or Pakistani rupee (PKR) 15000 in 2010 exchange rate terms). This information was collected from www.data.worldbank/indicator.

^13^
See Table A4 for descriptive statistics of variables in Equation (1).

^14^

*p*‐value for joint significance of (i) proportion of women who received prenatal checkup, (ii) proportion of women who received postnatal checkup, (iii) proportion of institutionalized births and, (iv) proportion of births attended by SBAs.

^15^

*p*‐value for joint significance of (i) proportion of rural households, (ii) average household income and, (iii) proportion of households own a TV.

^16^

*p*‐value for joint significance of (i) proportion of women who received prenatal checkup, (ii) proportion of women who received postnatal checkup, (iii) proportion of institutionalized births and, (iv) proportion of births, (v) proportion of rural households, (vi) average household income and, (vii) proportion of households own a TV.

^17^
Note that results in Table 3 control for district fixed effects. Since residuals were predicted at the district level, I cannot add them as an explanatory variables when district fixed effects are controlled for. Results in Table 3 and Table A5 are qualitatively similar but the magnitude of the impact is smaller with district fixed effects.

^18^
See https://www.unfpa.org/news/pakistan‐empowering‐midwives‐empower‐women for example, of such clinics.

^19^
Technical Resource Facility (2013) estimates the cost of training and deploying a community midwife in Pakistan at PKR 200,000 and PKR 270,000 respectively. The cost of each delivery is PKR 3750. These are converted into USD based on the exchange rate in 2013.

^20^
Household Income is adjusted for inflation over the different years of the surveys and reported in 2010 terms.

## APPENDIX A

**TABLE A1:** Pakistan social and living measurement survey sample size

Survey year	HH's interviewed	Sample size for this study
2004–05	74,420	30,986
2006–07	73,953	23,107
2008–09	75,188	29,600
2010–11	76,546	26,129
2012–13	77,764	28,845
2014–15	78,635	31,692

*Note*: Each survey round was conducted in all districts of Pakistan, barring districts with security issues. The survey years used in this study and shown in the table are those for which the data is representative at the district level. HH's interviewed indicates the overall number of households surveyed in each year of PSLM. This study uses outcomes for women who had given birth in 3 years prior to the time of survey and had answered the questions related to maternal health care, shown under the “sample size for the study” column.

Abbreviation: HH, household.

**TABLE A2:** Community midwives per 10,000 of the population in districts of Pakistan

Punjab		Sindh		Balochistan		KPK	
Lahore	0.21	Khairpur	0.36	Quetta	0.41	Swat	0.32
Attock	0.11	Sukkhur	0.18	Qillah Abdullah	0.24	Upper Dir	0.31
Rawalpindi	0.25	Karachi	0.02	Chaghi	0.10	Lower Dir	0.33
Jhelum	0.15	Nowsheroferoz	0.08	Sibbi	0.38	Chitral	0.26
Chakwal	0.14	Mirpurkhas	0.12	Khuzdar	0.28	Shangla	0.26
Sargodha	0.12	Jacobabad	0.04	Ketch/Turbat	0.38	Malakand	0.43
Khushaab	0.12	Shikarpur	0.04	Gwadar	0.46	Bonair	0.25
Bhakkar	0.14	Larkana	0.21	Loralai	0.15	Peshawer	0.45
Mianwali	0.23	Dadu	0.35	Qilla Saifullah	0.15	Charsada	0.31
Faisalabad	0.03	Hyderabad	0.20	Mastung	‐‐	Nowshera	0.21
Jhang	0.17	Badin	0.08	Awaran	‐‐	Kohat	0.18
T.T Singh	0.16	Thatta	0.08	Lasbela	‐‐	Karak	0.41
Gujranwala	0.10	Sanghar	0.08	Bolan	‐‐	Hangu	0.21
Gujrat	0.17	Thaparkar	‐‐	Zhob	‐‐	D.I.Khan	0.32
Sialkot	0.28	Nawabshah	‐‐	Kharan	‐‐	Lakki Marwat	0.32
Hafizabad	0.08	Ghotki	‐‐	Washuk	‐‐	Mansehra	0.17
Mandi Bahaddin	0.26			Barkhan	‐‐	Abbotabad	0.51
Narowal	0.50			Musakhel	‐‐	Haripur	0.29
Kasur	0.08			Nasirabad	‐‐	Bannu	0.20
Okara	0.17			Jafarabad	‐‐	Mardan	0.24
D.G.Khan	0.26			Jhal Magsi	‐‐	Swabi	0.26
Rajanpur	0.20			Pashin	‐‐	Tank	‐‐
Layyah	0.19			Dera Bugti	‐‐	Batgram	‐‐
Muzafargarh	0.17			Kalat	‐‐	Kohistan	‐‐
Bahawalpur	0.13			Ziarat	‐‐		
Shiekhupura	0.19			Kohlu	‐‐		
Vehari	0.36						
Multan	0.24						
Khanewal	0.15						
Lodhran	0.44						
Bahwalnagar	0.17						
R Y Khan	0.13						
Sahiwal	0.28						
Pakpattan	0.43						

*Note*: District boundaries in Pakistan changed over the years of survey used in this study. For consistency, I reassign the number of CMWs and the population of new districts to their parent districts. The list shown in this table is the final lists of districts used after reassignment to parent districts. The statistics shown are the average number of CMWs in each district in the post period.

**TABLE A3:** Description of variables

Variable	Description
Prenatal checkup	= 1 if the individual got at least one pre‐natal check‐up during pregnancy, 0 otherwise
Skilled attendance at birth	= 1 if the individual was assisted by a skilled birth attendant at time of birth, 0 otherwise
Institutionalized birth	= 1 if the individual gave birth at a medical facility, 0 otherwise
Post‐natal checkup	= 1 if the individual got at post‐natal checkup within 6 weeks of birth, 0 otherwise
CMW per 10,000	Number of CMWs per 10,000 of the district population
Region	= 1 if the individual resides in an urban area, 0 otherwise
LHW	= 1 if the household received a visit from a lady health worker in last 30 days
Time taken to reach the nearest health facility	Dummy variable for each of the following categories: 0–14 min, 15–29, 30–44, 45–59, more than 60 min
HH head education	Education of the head of the household (in years)
HH income	Total monthly income of the households (in 000s PKR)
HH members	Number of members in the household
TV ownership	= 1 if the household owns a TV, 0 otherwise
House ownership	= 1 if the household owns the house it resides in, 0 otherwise

*Note*: Variables defined are from the individual level data.

Abbreviations: CMW, community mid‐wives; HH, household; LHW, lady health worker; PKR, Pakistani rupee; TV, television.

**TABLE A4:** Descriptive Statistics for variables used to test endogeneity of the treatment variable

	Mean	Std. Dev.	Min.	Max.
Proportion of rural HHs in the district	0.479	0.101	0.141	0.840
Average HH income (PKR)	12,162.31	2460.42	8013.22	27,287.32
Proportion of births with skilled attendance at birth	0.340	0.167	0.033	0.824
Proportion of women who had a post‐natal check up	0.211	0.101	0.027	0.648
Proportion of institutional births	0.241	0.144	0.015	0.726
Proportion of women who had a pre‐natal check up	0.463	0.168	0.101	0.869
Proportion of HHs that own a TV	0.437	0.195	0.059	0.868
Proportion of HHs that received a LHW visit within the last 30 days	0.443	0.190	0.066	0.783

*Note*: These statistics are from the district level data aggregated from the individual micro data for the pre‐period years and used to estimate Equation (1). The aggregation yields one observation per district (*N* = 99).

Abbreviations: HH, household; LHW, lady health worker; PKR, Pakistani rupee; TV, television.

**TABLE A5:** Impact of Community Mid‐Wives (CMWs) on Maternal Health Care Utilization (with residuals, excluding fixed effects)

	1	2	3	4
	Pre‐natal	Post‐natal checkup	SBA	Inst. Birth
Panel A: Probit Estimation (with residuals added as a control)				
CMW per 10k	0.0957	0.294	0.577***	0.521***
	(0.197)	(0.237)	(0.151)	(0.139)
Residuals	−0.128	−0.501	−0.455	−0.562
	(0.318)	(0.331)	(0.372)	(0.394)
Panel B: Marginal Effects				
CMW per 10k	0.032	0.091	0.192***	0.171**
	(0.067)	(0.074)	(0.053)	(0.046)
Mean of the outcome	0.58	0.26	0.44	0.36
Observations	170,359	170,359	170,359	170,359

*Note*: Sample is women who had given birth in the last 3 years prior to the survey. Panel A shows results from probit estimation of Equation (2) ($\beta_{1}$ and $\beta_{4}$). CMWs per 10,000 of the population in the district is the treatment and “residuals” are predicted residuals obtained from Equation (1). Panel B shows the corresponding marginal effects from the estimations in Panel A. All estimations have the following controls: age dummies, education of the household head, real household income, number of members in the households, indicators for wealth of the household, indicator for rural/urban locality, indicators for time to nearest health facility, and survey year fixed effects. SBA stands for “skilled birth attendant”. Standard errors are clustered by district.

****p* < 0.001, ***p* < 0.01, **p* < 0.05.

## APPENDIX B ^21^

The CMW model includes the following functions:

- Providing individualized care to the pregnant women throughout the maternity cycle and the newborn, in her own environment and helping her in self‐care.
- Monitoring the physical, social and emotional well‐being of the pregnant woman as needed.
- Taking appropriate action within the resources available.
- Providing guidance and counseling to the community for healthy habits, and involving the family in preparation for childbirth and for unforeseen emergencies.
- Identifying actual or anticipated conditions requiring medical attention and making timely referrals.
- Practicing midwifery within the legal framework and following the professional code of conduct provided by the relevant authority.

The objectives of the program are defined as follows:

- To strengthen the existing midwifery and Lady Health Visitor training schools in the country through development of linkages with District Head Quarter and Tehsil Head Quarter hospitals for practical training, renovation of existing facilities and creation of domiciliary midwifery linkages.
- To improve the quality of midwifery training in the country through implementation of a standardized curriculum approved by the Pakistan Nursing Council, training and deployment of specific midwifery tutors and provision of standard training aids.
- To train a core group of master trainers at the National level for the training of midwifery tutors.
- To train 600 midwifery tutors all over Pakistan on the new curriculum for training of community midwives.
- To recruit, train and deploy 12,000 community midwives in the community by the end of the fourth year of the project.
- To promote birth preparedness activities through counseling.
- To strengthen the registration and accreditation system of the Pakistan Nursing Council through supporting additional staff for quality assurance and monitoring of trainings.
- Development of a computerized database of midwives at provincial MNCH directorates.

The proposed network of community midwives at the grass root level would play a pivotal role in achieving the following:

- Increasing the proportion of skilled birth attendants will have a definite impact on reducing maternal and newborn mortality and morbidity through:
- Establishing linkages, conducting normal deliveries at the household level and is able to recognize complications and takes decision of in‐time referrals of complicated obstetric cases to the higher level of functional health facilities.
- Delivery of quality RH‐PHC services at the community level.
- Established community midwifery schools with regards to human resource and equipment, thus providing a continuum of technical back stopping to the skilled birth attendants.
- LHWs Support provided through a regular contact with the SBAs would be established by the National Program for Family Planning and Primary Health Care through the LHWs supervisors.
- Including a socio‐culture change in the communities by counteracting the misinformation based on taboos and negative traditional practices.
- Provide opportunities for gender development and income generation thus helping poverty alleviation.
- Gradual replacement of TBAs by trained community midwives.

Selection Criteria

- Female, preferably married.
- Permanent resident of the area, for which she is applying.
- Minimum qualification should be at least Matric preferably with Science subjects obtaining 45% marks
- Have experience of working in the community
- Should be 18–35 years of age
